# Resveratrol enhances the chemotherapeutic response and reverses the stemness induced by gemcitabine in pancreatic cancer cells via targeting SREBP1

**DOI:** 10.1111/cpr.12514

**Published:** 2018-10-19

**Authors:** Cancan Zhou, Weikun Qian, Jiguang Ma, Liang Cheng, Zhengdong Jiang, Bin Yan, Jie Li, Wanxing Duan, Liankang Sun, Junyu Cao, Fengfei Wang, Erxi Wu, Zheng Wu, Qingyong Ma, Xuqi Li

**Affiliations:** ^1^ Department of Hepatobiliary Surgery First Affiliated Hospital, Xi'an Jiaotong University Xi'an China; ^2^ Department of Anesthesiology First Affiliated Hospital, Xi'an Jiaotong University Xi'an China; ^3^ Department of Neurosurgery Neuroscience Institute, Baylor Scott and White Health Temple Texas; ^4^ Neuroscience Institute, Baylor Scott & White Health Temple Texas; ^5^ Department of Surgery Texas A & M University Health Science Center, College of Medicine Temple Texas; ^6^ Department of Neurology Baylor Scott & White Health Temple Texas; ^7^ Department of Pharmaceutical Sciences Texas A & M University College of Pharmacy College Station Texas; ^8^ Department of General Surgery First Affiliated Hospital, Xi'an Jiaotong University Xi'an China

## Abstract

**Objectives:**

Gemcitabine is a standard treatment for advanced pancreatic cancer patients but can cause chemoresistance during treatment. The chemoresistant cells have features of cancer stem cells (CSCs). Resveratrol has been reported to overcome the resistance induced by gemcitabine. However, the mechanism by which resveratrol enhances chemosensitivity remains elusive. Here, we explored the mechanism by which resveratrol enhanced chemosensitivity and the role of sterol regulatory element binding protein 1 (SREBP1) in gemcitabine‐induced stemness.

**Materials and methods:**

The pancreatic cancer cell lines MiaPaCa‐2 and Panc‐1 were treated under different conditions. Methyl thiazolyl tetrazolium and colony formation assays were performed to evaluate effects on proliferation. Flow cytometry was conducted to detect apoptosis. Oil red O staining was performed to examine lipid synthesis. The sphere formation assay was applied to investigate the stemness of cancer cells. Immunohistochemistry was performed on tumour tissue obtained from treated KPC mice.

**Results:**

Resveratrol enhanced the sensitivity of gemcitabine and inhibited lipid synthesis via SREBP1. Knockdown of SREBP1 limited the sphere formation ability and suppressed the expression of CSC markers. Furthermore, suppression of SREBP1 induced by resveratrol reversed the gemcitabine‐induced stemness. These results were validated in a KPC mouse model.

**Conclusions:**

Our data provide evidence that resveratrol reverses the stemness induced by gemcitabine by targeting SREBP1 both in vitro and in vivo. Thus, resveratrol can be an effective chemotherapy sensitizer, and SREBP1 may be a rational therapeutic target.

## 1 INTRODUCTION

Pancreatic cancer (PC) is one of the top five leading causes of cancer‐related death^1^ and is predicted to take second place in 2030.^2^ Although numerous studies have been performed, the curative effect for PC is still not optimistic. Surgery is the preferred treatment for patients at the early stage. For those at the advanced stage, chemotherapy may be a choice. However, chemotherapeutic drugs can barely reach the tumour due to lack of blood supply and richness of mesenchymal components and desmoplasia. Currently, gemcitabine and FOLFIRINOX are recommended as first‐line treatments for PC patients diagnosed at the advanced stage. Generally, two types of chemoresistance can occur: intrinsic resistance and acquired resistance.^3^ The first type of chemoresistance occurs when treatment is invalid at the very beginning, and the second type occurs after several rounds of treatment with anticancer drugs. Nevertheless, chemoresistance can still occur even when little of the drug reaches the tumour.

Cancer stem cells (CSCs) are a subtype of cells with differentiative and self‐renewal capacities that result in cancer progression.^4^ CSCs are reported to be responsible for tumour initiation, progression, relapse, and chemoresistance.^4^, ^5^ Based on existing studies, CD44^+^/CD24^+^/ESA^+^ and CD133^+^ cells are considered pancreatic CSCs.^4^, ^6^, ^7^, ^8^, ^9^ Different pathways have been shown to be related to CSCs, including the PI3K/Akt, NOTCH, Wnt/β‐catenin, and NF‐κB signalling pathways.^8^, ^10^ Additionally, some miRNAs have been shown to regulate CSCs.^4^, ^11^ The existence of CSCs has been linked to chemoresistance^9^, ^12^, ^13^ and accumulating evidence indicates that CSCs may serve as indicators for tumour detection and be targets for cancer treatment.^7^, ^10^

Gemcitabine is a deoxycytidine analogue that is widely used for chemotherapy in various solid tumours.^14^ Since the overall survival of PC patients treated with gemcitabine has been shown to be superior to that of patients treated with 5‐fluorouracil, gemcitabine has become the standard treatment for PC patients.^3^ However, although gemcitabine exhibits anticancer activity, the effect of gemcitabine treatment on PC patients is not always good. The drug barely reaches the cancer, which subsequently leads to gemcitabine resistance.^3^, ^15^ However, the definitive mechanism of chemotherapy resistance is unknown. Pancreatic stellate cells, fibroblasts, microvesicles, immune cells, and CSCs have been reported to be involved in resistance to gemcitabine.^16^ In PC, CSCs display enhanced chemoresistance, whereas chemotherapy in turn promotes stemness.^17^, ^18^, ^19^

Resveratrol, which is a natural agent with an anticancer capacity, is found in various plants, including hellebore, grapes, berries, and peanuts, and belongs to the polyphenolic phytoalexins.^20^, ^21^ Resveratrol has been proven to be beneficial for the cardiovascular system and good for metabolic diseases.^22^ It has also been found to have anticancer activities in various cancer models, including renal cell carcinoma and PC, via different signalling pathways.^23^, ^24^, ^25^, ^26^, ^27^ Our previous studies demonstrated that resveratrol suppressed the EMT of PC cells by activating PI3K/Akt/NF‐κB^28^ and inhibited the invasive and migratory abilities via the Hedgehog signalling pathway.^29^ Moreover, we found that resveratrol activated the AMPK signalling pathway and suppressed YAP expression, which in turn enhanced sensitivity to gemcitabine.

Sterol regulatory element binding proteins (SREBPs) are members of a transcription factor family that control the expression of genes important for the uptake and synthesis of cholesterol, fatty acids, and phospholipids.^30^ Three isoforms of SREBPs (SREBP‐1a, SREBP‐1c, and SREBP‐2) are encoded by SREBF‐1 and SREBF‐2 respectively.^30^, ^31^, ^32^ Early studies focused on the effect of SREPBs on lipid metabolism, whereas recent studies explored the role of SREBPs in cancer. Increasing evidence indicates that SREBP1 motivates tumour progression, whereas suppression of SREBP1 may inhibit tumour growth.^31^, ^33^, ^34^ We previously found that inhibition of SREBP1 definitely restrained the proliferation of PC cells.

Here, we hypothesize that resveratrol inhibits lipid synthesis in PC cells and enhances the apoptosis induced by gemcitabine by blocking SREBP1 expression in PC cells. Moreover, resveratrol suppresses the gemcitabine‐induced stemness of PC both in vitro and in vivo. Our study indicates that inhibition of SREBP1 by resveratrol can be exploited as a novel target for chemoresistance correlated with CSCs in PC.

## 2 MATERIALS AND METHODS

All experimental protocols were approved by the Ethical Committee of the First Affiliated Hospital, Xi'an Jiaotong University, Xi’ an, China.

### 2.1 Materials and reagents

Resveratrol (>99% pure) was purchased from Sigma‐Aldrich (St. Louis, MO, USA), and gemcitabine was purchased from Selleck Chemicals (Houston, TX, USA). Resveratrol and gemcitabine were dissolved in DMSO to create 50 and 10 mmol/L stock solutions, respectively, and stored at −20°C. The working dilutions were freshly prepared in culture medium prior to use, and DMSO was used as the vehicle control. Antibodies targeting SREBP1, FASN (fatty acid synthase), PCNA (proliferating cell nuclear antigen), and β‐actin were purchased from Santa Cruz Biotechnology (Santa Cruz, CA, USA), and antibodies against Bax, Sox2, Nanog, and Oct4 were purchased from Abcam (Cambridge, UK). Other reagents were purchased from common commercial sources.

### 2.2 Cell culture

The human PC cell lines MiaPaCa‐2 and Panc‐1 were obtained from the Type Culture Collection of the Chinese Academy of Sciences (Shanghai, China). The cells were cultured in DMEM (Gibco, Grand Island, NY, USA) containing 10% foetal bovine serum (HyClone, Logan, UT, USA), penicillin G (100 U/mL), and streptomycin (100 g/mL) (Gibco) in a humidified atmosphere of 5% CO_2_ at 37°C.

### 2.3 Apoptosis assay

Pancreatic cancer (PC) cell apoptosis was assessed using flow cytometry with an Annexin V‐FITC/7‐AAD apoptosis detection kit purchased from Becton Dickinson and Company (BD) (Franklin Lakes, NJ, USA) according to the manufacturer's instructions. The preprocessing of the PC cells was performed as previously described.^35^ Then, the percentage of apoptotic cells was quantified by flow cytometry using a FACSCalibur flow cytometer (BD Biosciences, San Diego, CA, USA). The total apoptosis rate was assessed by adding the rates of the Annexin V‐FITC+/7‐AAD‐ (early apoptotic cells) and Annexin V‐FITC+/7‐AAD+ (late apoptotic cells) populations together.

### 2.4 Western blotting analysis

MiaPaCa‐2 and Panc‐1 cells with different treatment were harvested in RIPA lysis buffer (Beyotime, Guangzhou, China) to extract total proteins. The proteins were separated by SDS‐PAGE and transferred to polyvinylidene fluoride membranes. Next, the membranes were blocked with 5% fat‐free milk diluted in Tris‐buffered saline‐Tween for 2 hours at room temperature. After blocking, the membranes were incubated with the primary antibodies at 4°C overnight and then incubated with the second antibodies at room temperature for 2 hours. At the end of the incubations, an enhanced chemiluminescence kit (Millipore, Darmstadt, Germany) was used to detect the immunoreactive bands, and images were captured with the ChemiDoc XRS imaging system (Bio‐Rad Laboratories, Hercules, CA, USA).

### 2.5 Cell viability assay

Cancer cell lines (MiaPaCa‐2 and Panc‐1) were seeded into 96‐well plates at a density of 5000 cells/well and treated with resveratrol (50 μmol/L), gemcitabine (5 μmol/L), or resveratrol plus gemcitabine for the designated time periods (24, 48, and 72 hours). Cell viability was assessed using the methyl thiazolyl tetrazolium assay. The absorbance was measured at 490 nm with a multifunction microplate reader (POLARstar OPTIMA; BMG, Offenburg, Germany).

### 2.6 Colony formation assay

A total of 1 × 10^3^ cells were seeded into a 35‐mm Petri dish, and resveratrol, gemcitabine, or the combination agents were added on the second day. After treatment for 48 hours, the media were replaced with drug‐free media to allow colony formation for 2 weeks. At the indicated time point, the colonies were fixed with 4% paraformaldehyde and then stained with 0.1% crystal violet solution, rinsed, and imaged.

### 2.7 Oil red O staining

Oil red O was purchased from Sigma‐Aldrich. After treating PC cells with the normal control, DMSO or resveratrol for 48 hours, oil red O staining was performed according to the manufacturer's instructions. The images were recorded under a light microscope (Nikon Eclipse Ti‐S, Tokyo, Japan) at 400× magnification. Propanediol was added, and the optical density was measured at 510 nm to quantify the results.

### 2.8 Immunofluorescence staining

After treatment, the cells were rinsed with PBS and fixed with 4% formaldehyde for 15 minutes at room temperature. Then, 0.3% Triton X‐100 was added to increase the membrane permeability. Next, the cells were blocked with 5% BSA dissolved in Triton X‐100 for 1 hour and then incubated with the primary antibody overnight at 4°C. The following day, the cells were incubated with a secondary antibody conjugated with green fluorescence for 1 hour at room temperature. Then, the nuclei were stained with DAPI for 15 minutes. Finally, the coverslips were placed on the slides, and the cells were visualized with a Zeiss Instruments confocal microscope at 400× magnification.

### 2.9 RNA interference

To knockdown SREBP1 expression, three SREBP1‐specific siRNAs and one negative control siRNA were designed and synthesized by GenePharm (Shanghai, China). The siRNAs were transfected into PC cells with Lipofectamine 2000 according to the manufacturer's instructions. The cells were used in subsequent experiments 24 hours after transfection.

### 2.10 Sphere formation assay

Cells cultured under different conditions were harvested. A total of 1 × 10^3^ cells were seeded into six‐well ultra‐low attachment plates and cultured in DMEM/F12 medium containing 2% B27, 10 ng/mL of EGF, and 10 ng/mL of FGF. After 7 days of culture, the numbers of spheres were counted.

### 2.11 Genetically engineered transgenic mice

LSL‐Kras^G12D/+^; Trp53^fl/+^; Pdx1‐Cre (KPC) mice were purchased from the Nanjing Biomedical Research Institute of Nanjing University, Nanjing, China. The breeding and genotyping of the KPC mice were performed as previously described.^36^

### 2.12 Immunohistochemistry

Tumour samples were removed after the mice were sacrificed and fixed with 4% paraformaldehyde. Then, the samples were embedded in paraffin and cut for immunohistochemical staining. Immunohistochemical (IHC) staining was performed with the SABC kit (Maxim, Fuzhou, China) according to the manufacturer's instructions. The procedures were previously described.^36^, ^37^

### 2.13 Statistical analysis

The data in this study are presented as the means ± standard deviations (SDs). Student's *t*‐test was used to compare two groups. Statistical analyses for multiple comparisons were performed using one‐way ANOVA followed by the LSD post hoc test with SPSS (SPSS 18.0; SPSS Inc., Chicago, IL, USA). *P* < 0.05 was deemed significant. Each experiment was performed at least three times.

## 3 RESULTS

### 3.1 Resveratrol increases the sensitivity of PC cells to gemcitabine

**Figure 1:**
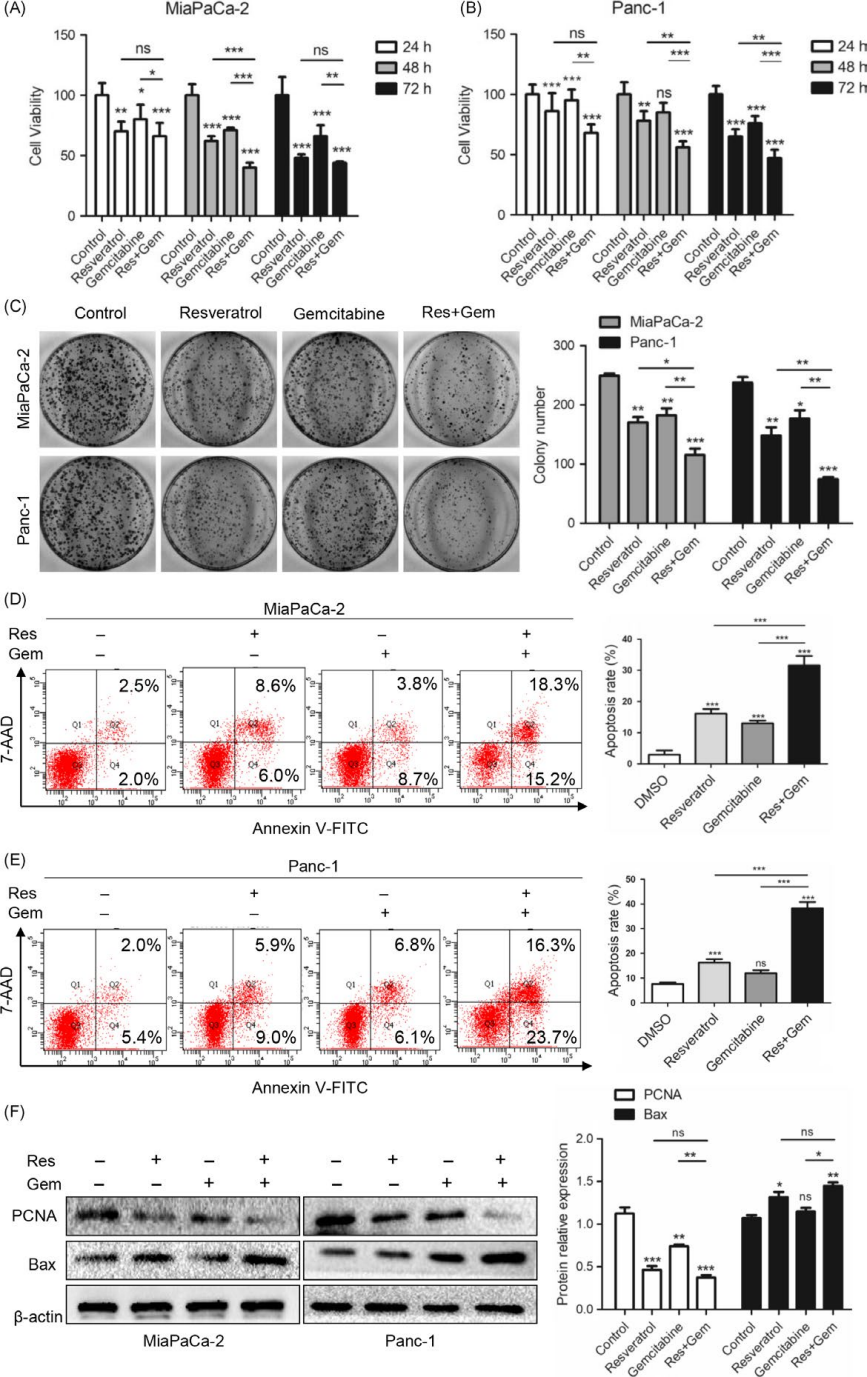
Resveratrol increases the sensitivity of pancreatic cancer (PC) cells to gemcitabine. (A, B) The effects of resveratrol (50 μmol/L) and gemcitabine (5 μmol/L) treatment on cell viability detected by the MTT assay. (C) The effects of resveratrol (50 μmol/L) and gemcitabine (5 μmol/L) treatment on the colony‐forming ability. (D, E) The effects of resveratrol (50 μmol/L) and gemcitabine (5 μmol/L) treatment on apoptosis of MiaPaCa‐2 and Panc‐1 cells detected by flow cytometry. (F) The effects of resveratrol (50 μmol/L) and gemcitabine (5 μmol/L) treatment on PCNA and Bax expression were detected by western blotting. The statistical graphs of the relative protein expression levels are shown. β‐actin was used as an internal loading control. ns, not significant, *P < 0.05, **P < 0.01, or ***P < 0.001

According to previous studies, gemcitabine induces apoptosis of PC cells, and resveratrol enhances the sensitivity of PC cells to gemcitabine.^27^ Here, we examined the effects of resveratrol (50 μmol/L) and gemcitabine (5 μmol/L) on the MiaPaCa‐2 and Panc‐1 cell lines. As shown in Figure 1A,B, resveratrol and gemcitabine treatment suppressed cell viability in a time‐dependent manner, with resveratrol plus gemcitabine showing a more significant effect. The colony formation assays showed that the colony numbers were significantly lower in the combination treatment group than in the resveratrol alone or gemcitabine alone groups (Figure 1C). As shown in Figure 1D,E, the apoptosis rates were increased significantly after treatment with resveratrol and gemcitabine together for 48 hours, which suggested that resveratrol facilitated the apoptosis induced by gemcitabine. Figure 1F shows that treatment with resveratrol and gemcitabine for 48 hours decreased PCNA and increased Bax expression, although resveratrol combined with gemcitabine was more effective. These results were consistent with those of our previous study.^27^ Together, these data indicate that resveratrol increases the sensitivity of PC cells to gemcitabine.

### 3.2 Resveratrol inhibits lipid synthesis and down‐regulates lipogenic genes

**Figure 2:**
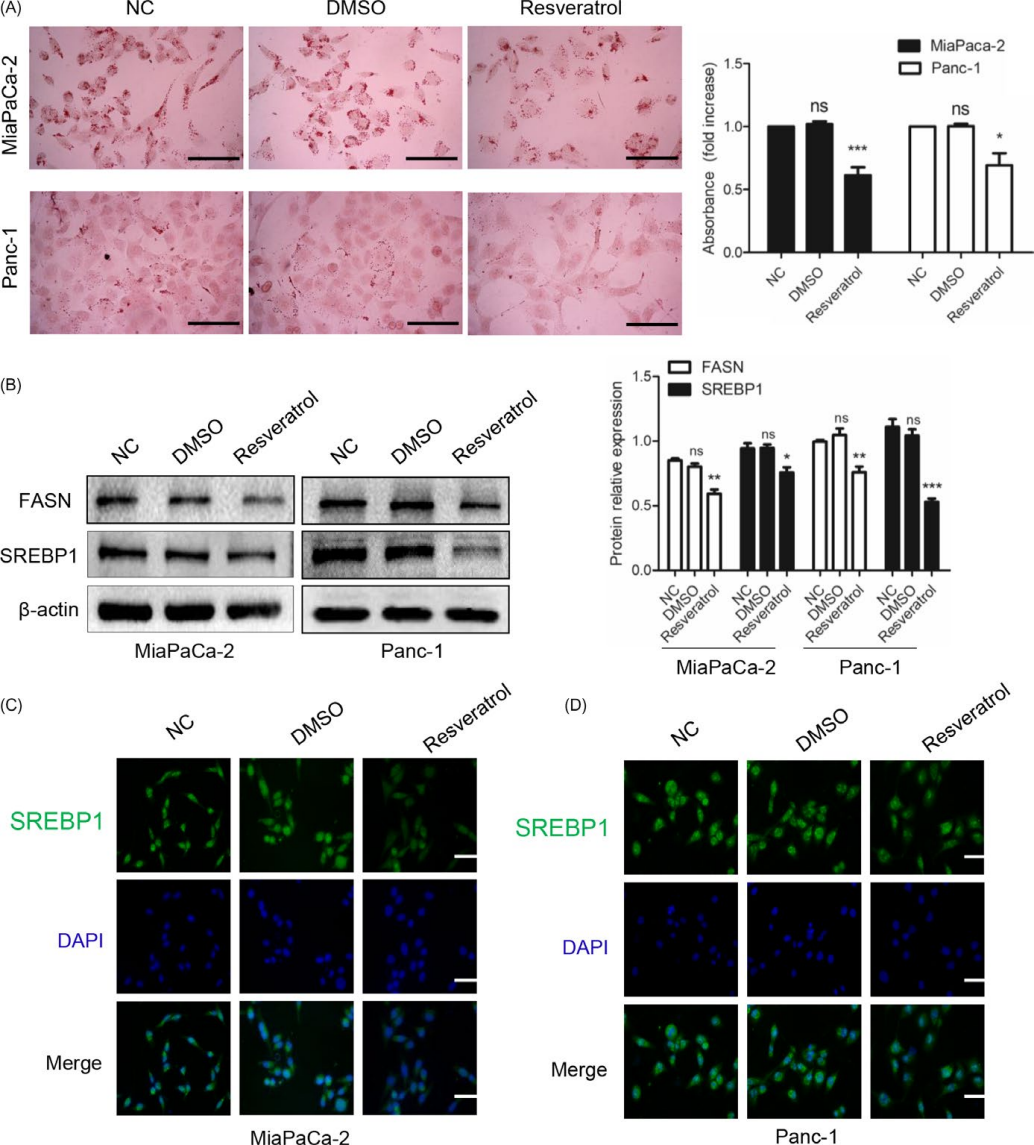
Resveratrol inhibits lipid synthesis and down‐regulates lipogenic gene expression. (A) After treatment with resveratrol (50 μmol/L) for 48 h, the lipid droplets of MiaPaCa‐2 and Panc‐1 cells were detected by oil red O staining. The magnification of the graph is 400×. Scale bars = 100 μm. The quantitative analysis is shown in the bar chart. (B) The effects of resveratrol on the FASN and SREBP1 expression levels were detected by western blotting. β‐actin was used as an internal loading control. (C, D) After resveratrol treatment (50 μmol/L), immunofluorescence staining was performed to evaluate the SREBP1 expression levels in MiaPaCa‐2 and Panc‐1 cells. SREBP1 staining is shown in green, and nuclear staining is shown in blue. The magnification is 400×. Scale bars = 100 μm. ns, not significant, *P < 0.05, **P < 0.01, or ***P < 0.001. Compared with the control group

Whether resveratrol has effects on lipid synthesis is unknown. To explore whether resveratrol affected lipid metabolism, we treated PC cells with resveratrol or not and then performed oil red O staining. As shown in Figure 2A, the number of lipid drops dramatically decreased after resveratrol treatment for 48 hours compared with the numbers in the normal control and DMSO‐treated groups. Quantitative analysis of oil red O staining was performed using a microplate reader (510 nm); the bar chart is shown in Figure 2A. Next, we examined FASN and SREBP1 expression, which are important components of the lipid synthesis process, using western blotting. Resveratrol treatment for 48 hours down‐regulated FASN and SREBP1 expression (Figure 2B). Furthermore, we performed immunofluorescence staining (Figures 2C,D) to verify the western blotting results. Similarly, we found that the green fluorescence representing SREBP1 was suppressed compared with the expression level in the control group. Thus, resveratrol inhibits lipid synthesis.

### 3.3 Knockdown of SREBP1 promotes gemcitabine sensitivity in PC cells

**Figure 3:**
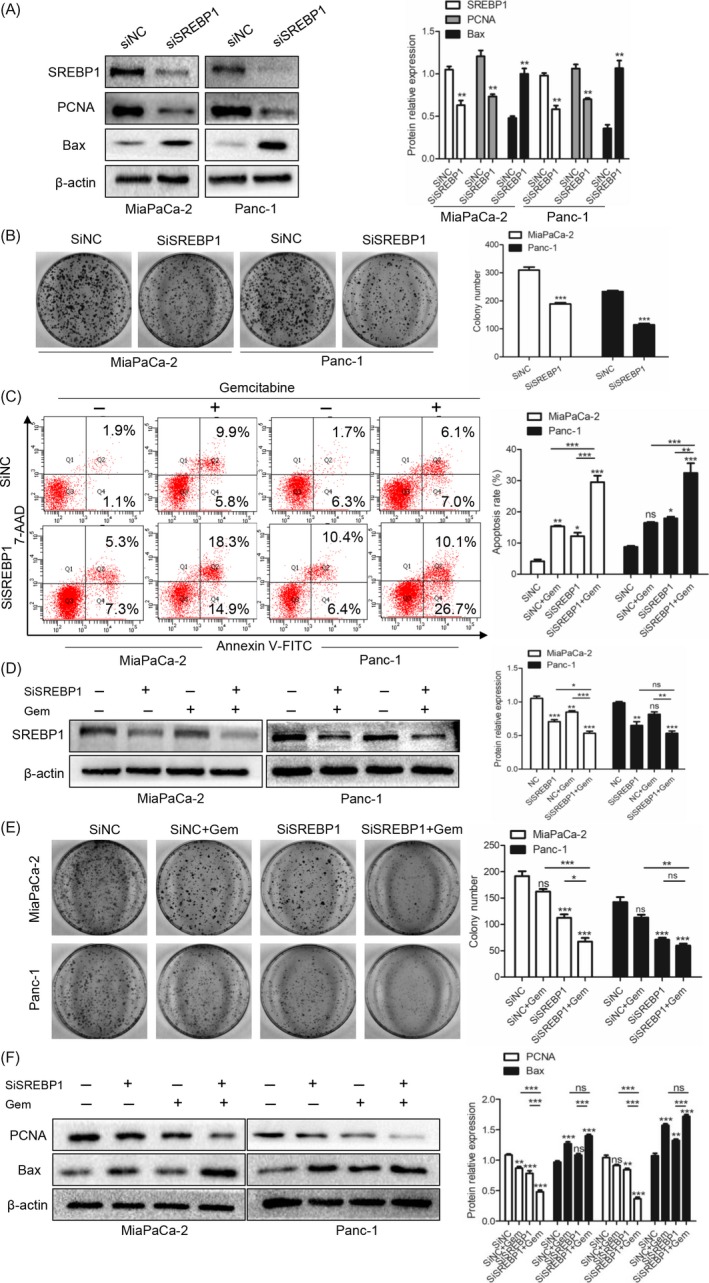
Knockdown of SREBP1 promotes gemcitabine sensitivity in pancreatic cancer (PC) cells. (A) The effects of SiNC and SiSREBP1 on the SREBP1, PCNA and Bax expression levels were detected by western blotting. **P < 0.01 compared with the control group. (B) Silencing SREBP1 suppresses the colony formation ability in PC cells. ***P < 0.001 compared with the control group. (C) The effects of SiSREBP1 and gemcitabine (5 μmol/L) treatment on apoptosis of MiaPaCa‐2 and Panc‐1 cells detected by flow cytometry. (D) The effects of SiSREBP1 and gemcitabine (5 μmol/L) treatment on the SREBP1 expression level in PC cells were detected by western blotting. (E) The effects of knockdown of SREBP1 on the colony formation ability in the MiaPaCa‐2 and Panc‐1 cell lines. (F) After knockdown of SREBP1 using SiSREBP1, the PC cell lines MiaPaCa‐2 and Panc‐1 were treated with gemcitabine (5 μmol/L). The PCNA and Bax expression levels were detected by western blotting. ns, not significant, *P < 0.05, **P < 0.01, or ***P < 0.001

To confirm that inhibition of SREBP1 increased the apoptosis induced by gemcitabine, we performed the following experiments. First, we used siRNA technology to knockdown SREBP1 and found that knocking down SREBP1 inhibited PCNA and promoted Bax expression (Figure 3A) and inhibited the colony formation ability (Figure 3B). Then, we evaluated PC cell apoptosis using flow cytometry. Knocking down SREBP1 increased the apoptosis induced by gemcitabine compared with the apoptotic cell numbers in the group treated with gemcitabine alone (Figure 3C). Then, we explored whether knockdown of SREBP1 plus gemcitabine decreased SREBP1 expression. As shown in Figure 3D, knocking down SREBP1 combined with gemcitabine treatment decreased SREBP1 expression and suppressed the colony formation ability (Figure 3E). Next, we detected the effect on proliferation and apoptosis by western blotting. As shown in Figure 3F, knocking down SREBP1 facilitated the effect of gemcitabine on proliferation and apoptosis. These data suggest that knocking down SREBP1 promotes gemcitabine sensitivity in PC cells.

### 3.4 Knockdown of SREBP1 suppresses the stemness and resveratrol rescues the stemness induced by gemcitabine via suppressing SREBP1

**Figure 4:**
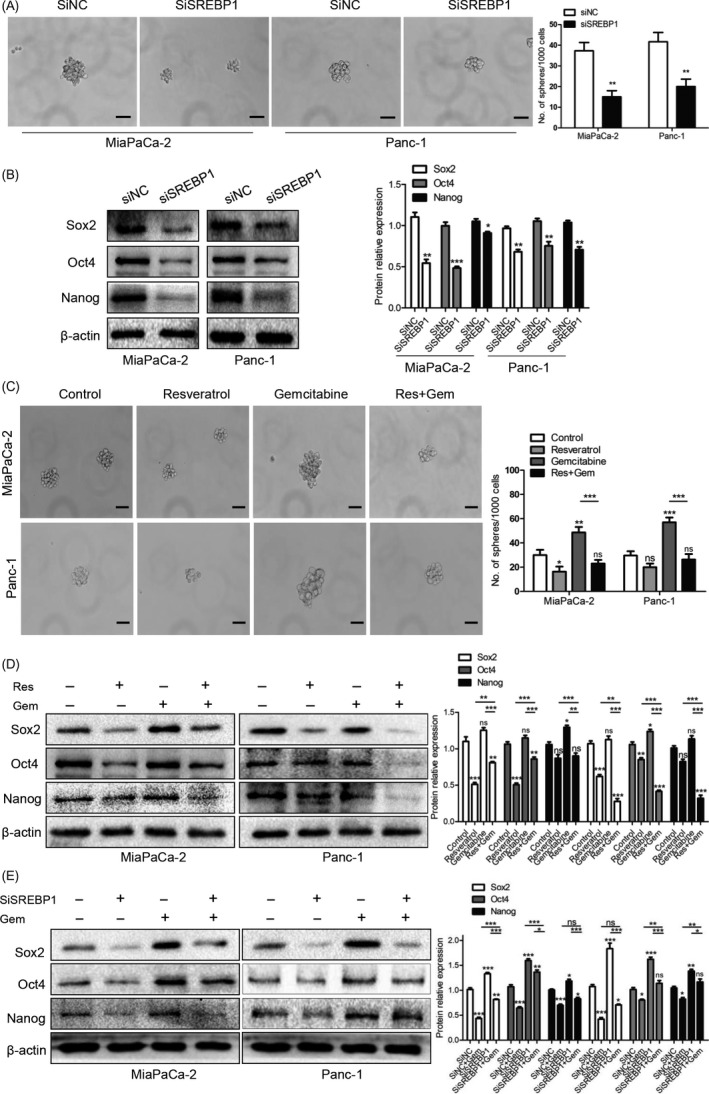
Knockdown of SREBP1 suppresses the stemness and resveratrol rescues the stemness induced by gemcitabine via suppressing SREBP1. (A) Sphere formation assays were performed after knockdown of SREBP1. The images were taken at a magnification of 200× on the 7th day. Scale bars = 50 μm. The numbers of sphere were counted. **P < 0.01 compared with the control group. (B) The Sox2, Oct4, and Nanog expression levels after knockdown of SREBP1 were detected by western blotting. *P < 0.05, **P < 0.01, or ***P < 0.001 compared with the control group. (C) After resveratrol (50 μmol/L) and gemcitabine (5 μmol/L) treatment for 48 h, pancreatic cancer (PC) cells were subjected to the tumour sphere‐forming assay. The sphere numbers of the MiaPaCa‐2 and Panc‐1 cells were counted. The magnification of the graph is 200×. Scale bars = 50 μm. (D) The effects of resveratrol (50 μmol/L) and gemcitabine (5 μmol/L) treatment on the Sox2, Oct4, and Nanog expression levels were detected by western blotting. (E) After treatment with SiSREBP1 combined with gemcitabine (5 μmol/L), western blotting was performed to detect the Sox2, Oct4, and Nanog expression levels in the MiaPaCa‐2 and Panc‐1 cell lines. ns, not significant, *P < 0.05, **P < 0.01, or ***P < 0.001

To investigate the effect of SREBP1 on the stemness of cancer cells, we performed sphere formation assays. Pancreatic cancer cells were treated with SiNC or SiSREBP1 and cultured for 7 days; then, the sphere‐forming ability of the cancer cells was determined. As shown in Figure 4A, the cells with knocked down SREBP1 exhibited a decreased ability to form spheres. Sox2, Nanog, and Oct4 are stem cell markers. Next, we performed western blotting to detect the expression levels of these stem cell markers; as shown in Figure 4B, all three markers were down‐regulated after knockdown of SREBP1, which was similar to the results obtained in the sphere formation assays.

We confirmed that resveratrol decreased SREBP1 expression and that knockdown of SREBP1 suppressed the sphere formation ability and Sox2, Nanog, and Oct4 expression in PC cells. To investigate the effect of resveratrol on the stemness induced by gemcitabine, we treated PC cells with either DMSO as a control, resveratrol alone, gemcitabine alone, or the combination of resveratrol and gemcitabine for 48 hours. As shown in Figure 4C, resveratrol decreased and gemcitabine promoted the sphere formation ability. Interestingly, PC cells treated with both resveratrol and gemcitabine exhibited a lower sphere formation ability than the cells treated with gemcitabine alone.

Next, we examined expression of the three stem cell markers. We found that the expression of all three markers was suppressed when the cells were treated with resveratrol alone and was up‐regulated when the cells were treated with gemcitabine alone; resveratrol and gemcitabine together rescued the expression levels of the stem cell markers induced by gemcitabine (Figure 4D). We also tested changes in pancreatic cancer cells treated with SiSREBP1 and gemcitabine. Pancreatic cancer cells treated with the SREBP1 siRNA and gemcitabine exhibited decreased levels of these stem cell markers compared with the cells treated with gemcitabine alone (Figure 4E). Together, our results show that knockdown of SREBP1 decreases the stemness of PC cells and that resveratrol rescues the stemness induced by gemcitabine via suppressing SREBP1.

### 3.5 Resveratrol inhibits gemcitabine‐induced stemness in vivo

**Figure 5:**
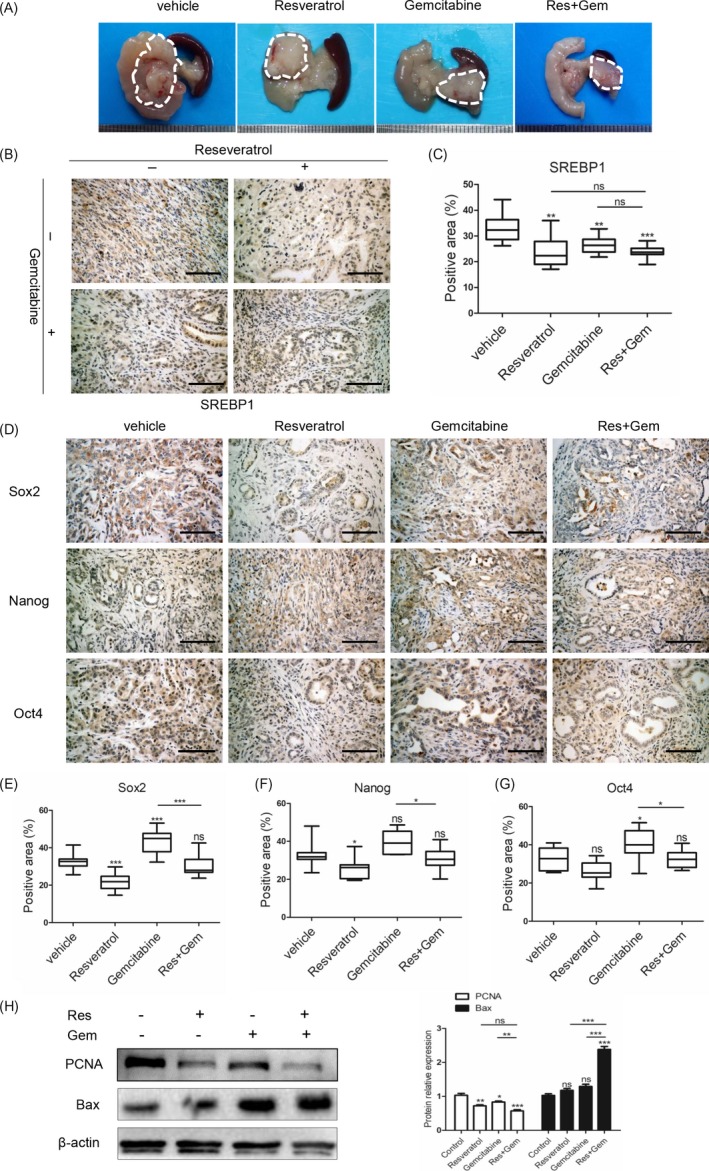
Resveratrol inhibits gemcitabine‐induced stemness in vivo. (A) Representative macroscopic images of PDAC in KPC mice in different treatment groups. The dotted line shows the tumour location. (B,C) The SREBP1 expression level was detected by immunohistochemistry, and the SREBP1‐positive area was measured in 10 random fields of view. Scale bars = 100 μm, (D) IHC pictures of Sox2, Oct4, and Nanog staining in the vehicle, resveratrol, gemcitabine, and resveratrol combined with gemcitabine groups. Scale bars = 100 μm. (E‐G) Statistical analysis of the Sox2‐, Oct4‐ and Nanog‐positive areas. (H) The PCNA and Bax protein expression levels in the extracted tumour tissues were detected by western blotting. ns, not significant, *P < 0.05, **P < 0.01, or ***P < 0.001

We assessed the effect of resveratrol on the suppression of gemcitabine‐induced stemness in the KPC mouse model. At 6 weeks of age, the mice were divided randomly into four groups with five mice per group. The mice in the control and resveratrol groups were treated daily with vehicle or resveratrol (50 mg/kg) by gavage respectively. The mice in the gemcitabine group were treated with gemcitabine (50 mg/kg) weekly by intraperitoneal injection, and the mice in the resveratrol plus gemcitabine group were treated with resveratrol (50 mg/kg) daily and gemcitabine (50 mg/kg) weekly. At 4 months of age, the mice were sacrificed by euthanasia. We collected the tumours and performed IHC. Figure 5A shows representative macroscopic images of the KPC mice in each group. As shown in Figure 5B,C, the mice treated with resveratrol, gemcitabine, or resveratrol plus gemcitabine had decreased SREBP1 expression in their tumour tissues compared with the expression levels in the mice treated with vehicle.

Then, we examined the expression levels of the stem cell markers. All markers were decreased after treatment with resveratrol, whereas treatment with gemcitabine up‐regulated expression of these markers. The stem cell markers in the tumours from the mice treated with resveratrol plus gemcitabine were expressed at high levels that were comparable to those in the mice from the vehicle group, which suggested that resveratrol rescued the stemness induced by gemcitabine (Figure 5D‐G). We extracted proteins from the tumour tissues and evaluated the PCNA and Bax expression levels by western blotting (Figure 5H). These results were in accordance with those obtained from the in vitro studies. Thus, our data indicated that resveratrol rescued the gemcitabine‐induced stemness both in vitro and in vivo.

## 4 DISCUSSION

In this study, we show that resveratrol inhibits lipid synthesis and enhances sensitivity to gemcitabine through SREBP1 suppression. Moreover, we demonstrate that resveratrol overcomes the gemcitabine‐induced stemness via suppressing SREBP1 both in vitro and in vivo.

Numerous studies found that gemcitabine inhibited proliferation and migration and induced apoptosis to suppress tumour growth in PC.^18^, ^19^ To improve the efficiency of chemotherapy, resveratrol, metformin, and ormeloxifene have been shown to enhance the efficacy of gemcitabine treatment.^27^, ^38^, ^39^ A previous study reported that resveratrol enhanced the sensitivity of PC cells to gemcitabine via activating the AMPK signalling pathway.^27^ Moreover, a study performed in nude mice determined the effect of resveratrol plus gemcitabine in vivo. According to that study, resveratrol potentiated the effect of gemcitabine on tumour growth.^40^ In our present study, we found that resveratrol enhanced the sensitivity of PC cells to gemcitabine via down‐regulating SREBP1 expression.

Although gemcitabine is used as the initial therapy for advanced PC, most patients develop resistance during the initial treatment period.^3^, ^41^ However, the definite mechanism of chemoresistance is unknown. A few recent reports showed that gemcitabine treatment fortified the stemness of cancer cells through the Nox/ROS/NF‐κB/STAT3 signalling cascade and the lncRNA HOTAIR.^19^, ^42^ Similarly, our data indicate that gemcitabine treatment promotes the sphere formation ability and up‐regulates CSC marker expression in the PC cell lines MiaPaCa‐2 and Panc‐1.

Metabolic reprogramming is thought to be a characteristic of cancers that makes cancer cells more adaptive to different environments.^43^, ^44^ Lipid synthesis is enhanced in PC cells, and suppressing the lipid synthesis process has been confirmed as a target for cancer treatment.^33^, ^34^, ^35^ As a key transcription factor during lipid synthesis, SREBP1 has received much attention. Inhibition of SREBP1 has been reported to suppress the proliferation, invasion, and migration of lung cancer, prostate cancer, renal cell carcinoma, glioblastoma, and PC.^31^, ^33^, ^45^, ^46^, ^47^ Some researchers demonstrated that enhanced lipid synthesis facilitated gemcitabine resistance through endoplasmic reticulum stress.^48^ In this study, we found that knockdown of SREBP1 by siRNA inhibited the stemness of PC cells. Meanwhile, down‐regulation of SREBP1 by resveratrol overcame the stemness induced by gemcitabine in both PC cell lines and the KPC mouse model.

Based on the results of our present study, we can conclude that resveratrol restrains lipid synthesis and suppresses the stemness induced by gemcitabine via down‐regulation of SREBP1. Overall, our data provide evidence that resveratrol enhances sensitivity to gemcitabine and reverses the stemness induced by gemcitabine via down‐regulating SREBP1 expression. These findings suggest that resveratrol is a potent chemotherapy sensitizer and that SREBP1 is a notable target for cancer treatment.

## CONFLICT OF INTEREST

The authors declare that they have no conflicts of interest with the contents of this manuscript.
